# Recent advances in two‐dimensional materials for hydrovoltaic energy technology

**DOI:** 10.1002/EXP.20220061

**Published:** 2023-01-28

**Authors:** Zhihang Liu, Chao Liu, Zhaotian Chen, Honglan Huang, Yifan Liu, Liang Xue, Jingwen Sun, Xin Wang, Pan Xiong, Junwu Zhu

**Affiliations:** ^1^ Key Laboratory for Soft Chemistry and Functional Materials of Ministry Education, School of Chemistry and Chemical Engineering Nanjing University of Science and Technology Nanjing China

**Keywords:** energy conversion efficiency, hydrovoltaic energy, output power, self‐powered devices, two‐dimensional materials

## Abstract

Hydrovoltaic energy technology that generates electricity directly from the interaction of materials with water has been regarded as a promising renewable energy harvesting method. With the advantages of high specific surface area, good conductivity, and easily tunable porous nanochannels, two‐dimensional (2D) nanomaterials have promising potential in high‐performance hydrovoltaic electricity generation applications. Herein, this review summarizes the most recent advances of 2D materials for hydrovoltaic electricity generation, including carbon nanosheets, layered double hydroxide (LDH), and layered transition metal oxides and sulfides. Some strategies were introduced to improve the energy conversion efficiency and the output power of hydrovoltaic electricity generation devices based on 2D materials. The applications of these devices in self‐powered electronics, sensors, and low‐consumption devices are also discussed. Finally, the challenges and perspectives on this emerging technology are outlined.

## Graphical abstract

Hydrovoltaic energy technology that generates electricity directly from the interaction of materials with water has been regarded as a promising renewable energy harvesting method. This review summarizes the most recent advances of 2D materials for hydrovoltaic electricity generation, including carbon nanosheets, layered double hydroxide, and layered transition metal oxides and sulfides. Perspectives toward the future applications of 2D materials in hydrovoltaic electricity generation are also outlined.

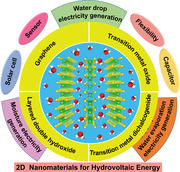

## 1 INTRODUCTION

With the accelerating depletion of traditional energy sources and the relevant environmental crisis, the utilization and exploration of renewable energy have attracted increasing attention.^[^ ^1^, ^2^, ^3^, ^4^ ^]^ As an emerging green energy technology, hydrovoltaic energy systems can obtain electricity by capturing the hydropower in the hydrologic cycle, including water evaporation electricity generation, moisture electricity generation, and water drop electricity generation, etc.^[^ ^5^, ^6^, ^7^, ^8^ ^]^ Compared with traditional renewable energy, hydrovoltaic energy can be liberated from geographical restriction and harvest energy directly from any environment which contains water.^[^ ^9^, ^10^ ^]^ During hydrovoltaic electricity generation, an electric voltage is generated when the electrolyte flow moves through a charged microporous channel under a pressure gradient. The output power is mainly related to the direct interaction between water and solid materials, which will cause the asymmetry of structural electronics and the nonuniform distribution of ions.^[^ ^11^, ^12^, ^13^ ^]^ This inspires the development and utilization of nanomaterials for hydrovoltaic electricity generation because the nanochannels with atomically smooth walls in nanomaterials are suggested to enhance the water–solid interactions.

Recently, various hydrovoltaic electricity generation devices based on nanomaterials have been investigated. For instance, Liu et al. found that plasma‐treated carbon nanoparticles could generate electricity under a moist environment.^[^ ^11^ ^]^ Zhou and co‐workers reported that the printable carbon black (CB) could generate electricity by evaporating natural water.^[^ ^14^ ^]^ Compared with other nanomaterials such as zero‐dimensional nanomaterials, electronic confinement in two‐dimensional (2D) nanomaterials endows them with stronger electronic properties, making them promising candidates for application of hydrovoltaic electricity generation. 2D nanomaterials, especially those atomically thin nanosheets, can expose all their surfaces, resulting in enhanced electric conversion efficiency. Due to these features, 2D nanomaterials are extremely sensitive to extrinsic water flow and can generate more substantial water–solid interactions. So far, numbers of 2D nanomaterials have been extensively investigated as one of the most promising candidates for the development of hydropower generators. For example, pristine graphene oxide (GO) films were reported to generate high voltages of 0.4–0.7 V under heated water vapor.^[^ ^15^ ^]^ Guo's team reported a voltage was induced by moving water droplets on a graphene strip.^[^ ^16^ ^]^ Zhao and co‐workers demonstrated that the induced voltage of GO films with a functional group gradient was about 0.26 V in a high humidity environment.^[^ ^8^ ^]^ However, there are still two main challenges for hydrovoltaic electricity generation: (1) the mechanism of hydrovoltaic electricity generation; (2) the low output power. Although some mechanisms have been proposed, they are still not perfect and cannot clearly explain the electricity generation process. The perfection of mechanisms still needs to be constantly explored in the future. The output power is mainly limited by relatively low ion concentration gradients and the large distances between ions and surfaces of the materials in previous reports. The two key points to improve the output power focus on accelerating the transportation of ions inside the materials and strengthening the interfacial interactions between materials and water molecules.^[^ ^17^, ^18^, ^19^, ^20^ ^]^ Structure engineering of 2D materials can enhance the output power efficiently by improving both the ion migration rates and the water–solid interface interactions. There are two main methods to accomplish the above targets: (1) adjustment of the micro‐nano structures of 2D materials and (2) increase of the surface potentials of 2D materials.^[^ ^12^, ^21^, ^22^, ^23^, ^24^, ^25^, ^26^ ^]^ In method one, the specific surface areas could be enlarged by adjusting the micro‐nanostructures of 2D materials, leading to improved absorption of water molecules on 2D materials. Therefore, the water–solid interface interactions could be obviously improved. For example, the GO was partially reduced to reduced graphene oxides (rGO) during an annealing treatment. Owing to the relatively high porosity and large specific surface areas, the resulting rGO sponges could generate a stable voltage by the evaporation of natural water.^[^ ^27^ ^]^ In method two, more free‐charged ions could be released at the water–solid interfaces due to the increased surface potential of solid materials. This will increase the distribution of free ions. Thus, the water–solid interactions will be enhanced effectively, resulting in improved output power. For example, Qu's group reported a gradient structure of graphene by reconfiguring the inner oxygen‐containing functional groups. The functional groups with concentration gradients showed different surface potential, facilitating charge migration, and inducing a current flow, thus outputting higher power.^[^ ^28^ ^]^ Based on the above advances, 2D nanomaterials have shown great potential in hydrovoltaic electricity generation. Although several preview reviews have summarized the development of nanomaterials for harvesting electrical energy from the environment,^[^ ^29^, ^30^ ^]^ a timely summary that focuses on 2D materials for hydrovoltaic energy technology has rarely been reported, to the best of our knowledge.

Herein, we summarize the recent advances of 2D materials in hydrovoltaic electricity generation. We first introduce hydrovoltaic electricity generation based on different water forms, moisture, water drop, and water evaporation. Then we focus on several typical 2D materials for hydrovoltaic electricity generation, including carbon nanosheets, layered double hydroxide (LDH), and layered transition metal oxides and sulfides. We also introduce the potential device applications of hydrovoltaic electricity generation for self‐powered devices and for powering socially desirable low‐consumption devices. Finally, we discuss the challenges and perspectives on this emerging technology for further research.

## 2 HYDROVOLTAIC ELECTRICITY GENERATION

**FIGURE 1:**
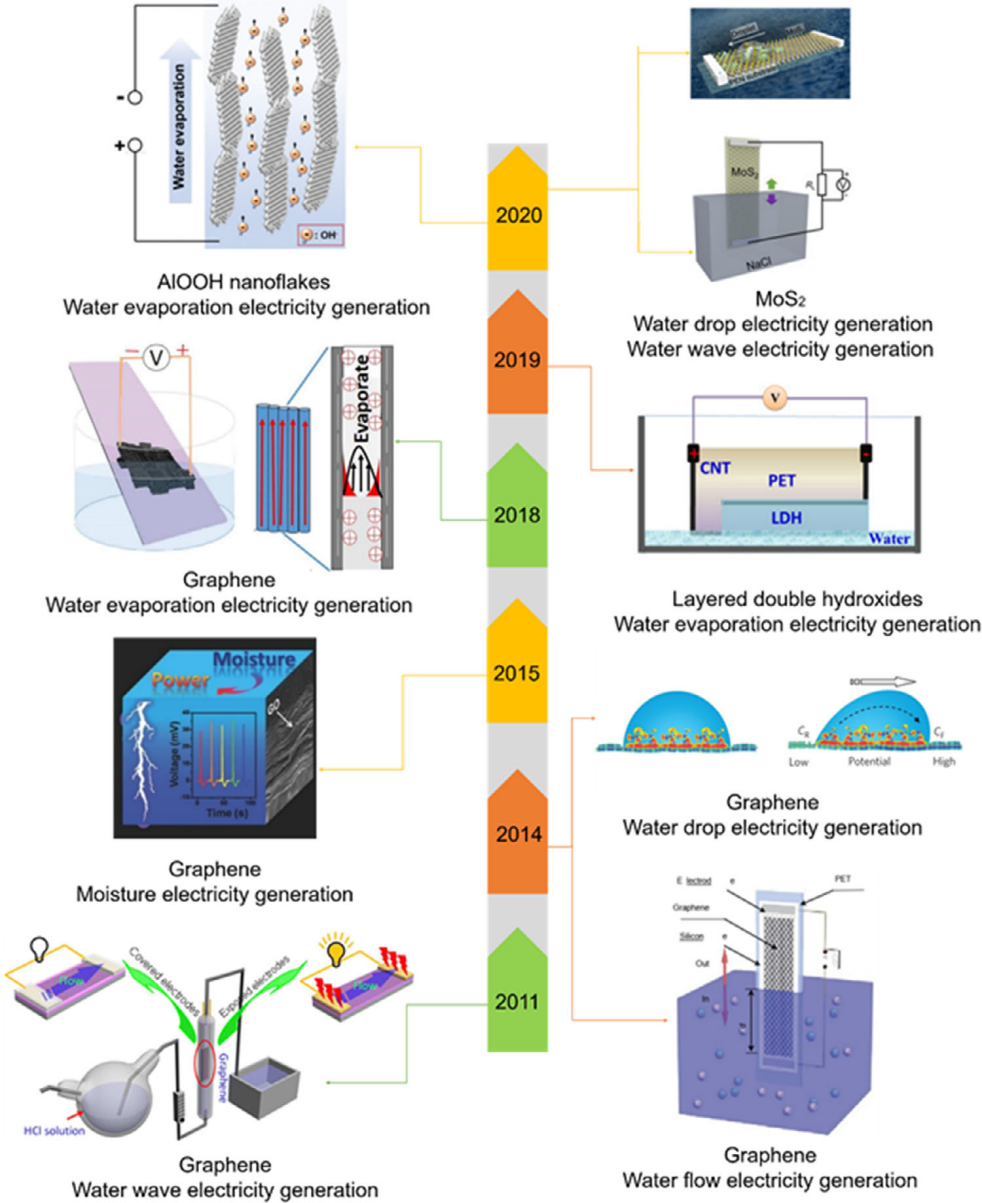
Development history of hydrovoltaic electricity generation. Timeline of the development of hydrovoltaic electricity generation. 2011: Adapted with permission.^[^ ^25^ ^]^ Copyright 2011, American Chemical Society. 2014: Adapted with permission.^[^ ^33^ ^]^ Copyright 2014, Nature Publishing Group. 2014: Adapted with permission.^[^ ^16^ ^]^ Copyright 2014, Nature Publishing Group. 2015: Adapted with permission.^[^ ^8^ ^]^ Copyright 2015, Wiley‐VCH. 2018: Adapted with permission.^[^ ^34^ ^]^ Copyright 2018, Elsevier. 2019: Adapted with permission.^[^ ^41^ ^]^ Copyright 2019, Elsevier. 2020: Adapted with permission.^[^ ^44^ ^]^ Copyright 2020, Elsevier. 2020: Adapted with permission.^[^ ^45^ ^]^ Copyright 2020, Wiley‐VCH.

Hydrovoltaic electricity generation is a technology that directly utilizes the interaction of materials and water.^[^ ^3^, ^31^ ^]^ Since discovering the phenomenon of hydrovoltaic electricity generation, there has been widespread interest in using 2D nanomaterials for this energy harvesting (Figure 1). As early as 2003, Sood and Ghosh et al. experimentally found that electrical signals were generated on carbon nanotubes in a flowing ionic liquid, which initiates the subsequent research on hydrovoltaic electricity generation.^[^ ^32^ ^]^ Later, Dhiman's group studied the interaction between 2D materials and water in hydrovoltaic electricity generation.^[^ ^25^ ^]^ They immersed graphene in a flowing dilute hydrochloric acid solution and found that it could generate a voltage several times larger than that of carbon nanotubes under the same conditions. In 2014, Guo and co‐workers achieved millivolt magnitudes of voltage via flowing liquid droplets on the surface of graphene.^[^ ^16^ ^]^ Meanwhile, they also found that the insertion and extraction motion of graphene into/out of the solution would generate a difference in potentials generated at both ends of the graphene.^[^ ^33^ ^]^ Furthermore, Qu et al. reported electricity generation from 2D materials in the moisture environment. They successfully used GO under exposure to moist environment to generate electricity in 2015 owing to the water adsorption capacity of GO.^[^ ^8^ ^]^ Recently, research on natural water evaporation‐induced electricity generation has also appeared. Zhou et al. discovered that the electricity could generate by water evaporation through CB sheets.^[^ ^5^ ^]^ In 2018, Hou et al. discovered that when a graphene film is immersed in a NaCl solution, a voltage of about 0.37 V can be generated as the solution evaporates.^[^ ^34^ ^]^ Since then, 2D materials such as GO,^[^ ^8^, ^28^, ^35^, ^36^, ^37^, ^38^, ^39^ ^]^ LDH,^[^ ^40^, ^41^ ^]^ oxides, and sulfides have been studied thoroughly in hydrovoltaic electricity generation.^[^ ^42^, ^43^, ^44^ ^]^

To harvest the hydrovoltaic electricity effectively, it is important to reveal the interactions between water and solid surfaces. When the solid surface is in contact with the liquid, due to the electrostatic attraction or the surface potential at the liquid‐solid interfaces, certain charged components in the liquid will be selectively adsorbed to form a tight adsorption layer on the solid surfaces. Then, the ions in the solution with opposite charges will be subjected to the Coulomb force and attracted to the vicinity of the solid‐liquid interface.^[^ ^5^, ^16^, ^20^ ^]^ Therefore, a region enriched by two layers of heterogeneous charged ions is formed at the interface as a whole, which is called the “Electric Double Layer” (EDL).^[^ ^20^, ^26^, ^46^ ^]^ EDL formation at the water–solid interface or the reaction between water molecules and functional groups on the solid surfaces can produce migrating ions, where the distribution of functional groups on the material surface plays a crucial role.^[^ ^29^, ^47^ ^]^

**FIGURE 2:**
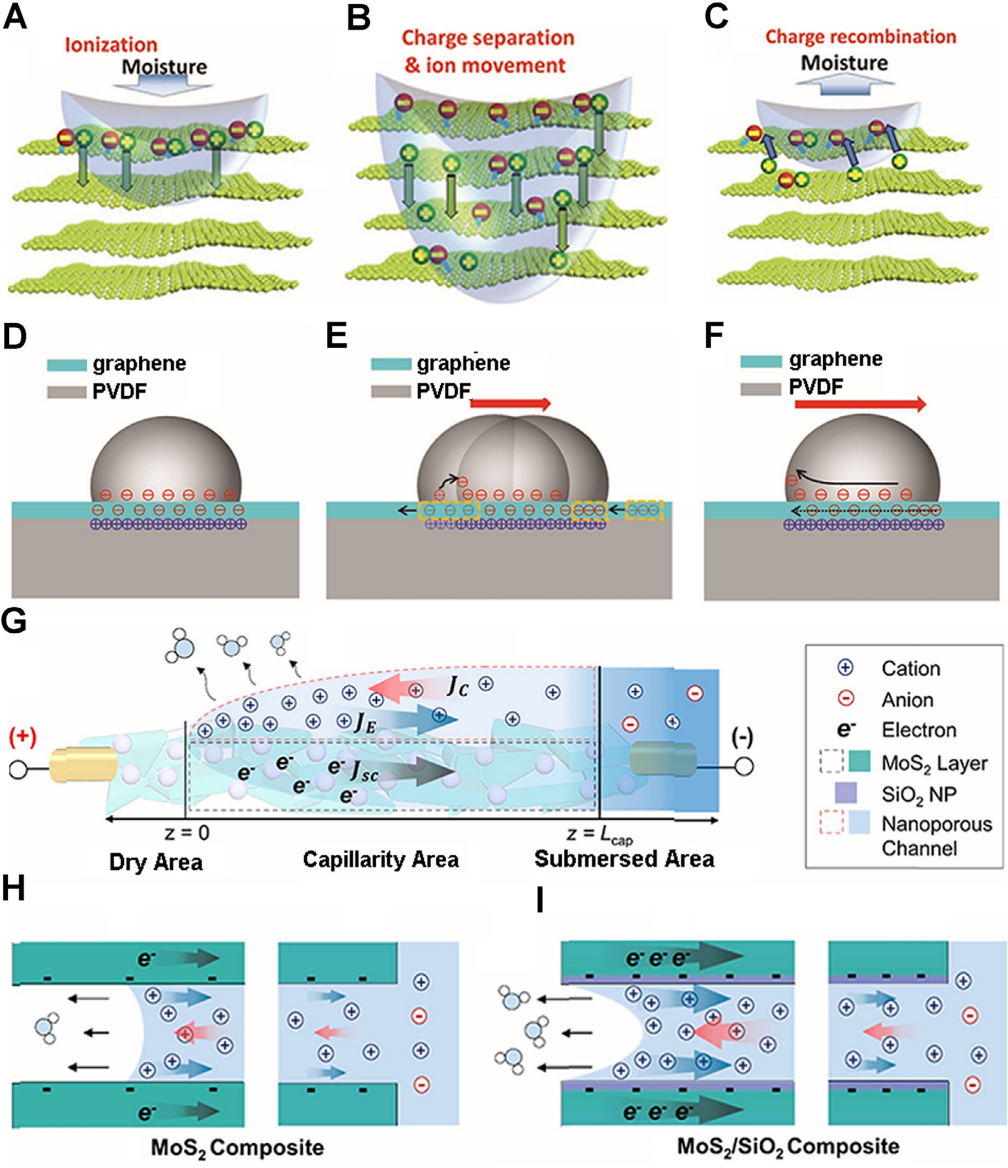
The mechanisms of hydrovoltaic electricity generation. (A–C) The dynamic evolution of the moisture electricity generation. Adapted with permission.^[^ ^15^ ^]^ Copyright 2018, Wiley‐VCH. (D–F) Schematic diagram of charge transfer with the flow of water droplets. Adapted with permission.^[^ ^49^ ^]^ Copyright 2017, Wiley‐VCH. (G) Schematic diagram of electricity generation induced by water evaporation. (H,I) The movement of carriers in a 2D material. Adapted with permission.^[^ ^43^ ^]^ Copyright 2021, Elsevier.

Based on the above theories, the mechanisms of hydrovoltaic electricity generation have been fully developed. According to the difference of the generated mechanism, hydrovoltaic electricity generation could be roughly divided into three categories, moisture electricity generation, water drop electricity generation, water evaporation electricity generation. The process of moisture electricity generation can be divided into three steps. Ionization is the first step in the whole process. When water molecules approach the oxygen‐containing functional groups on the surfaces of nanomaterial, the locally ionized nanomaterials will release many free H^+^ ions (Figure 2A).^[^ ^8^, ^37^, ^38^ ^]^ Second, the untrammeled H^+^ ions will move along the interlamellar spacing with water molecules (Figure 2B). Finally, the desorption process will occur when sufficient protons are released, resulting in a drop in potential (Figure 2C). The fundamental mechanism for the water drop power generation is the formation and disappearance of EDL at the head and tail parts of the droplets during the advancement motion. Besides, as long as the droplet is continuously flowing, this charge transfer process can be maintained steadily between water and the surfaces of substrates. The ions are first attracted to the front end of the water drop and then released at the rear end, generating a potential difference between both ends of the droplet (Figure 2D–F).^[^ ^16^, ^48^, ^49^ ^]^ The mechanism of water wave electricity is similar to water drop electricity generation. As the material moves in and out of the solution, a similar process of EDL formation and disappearance occurs, resulting in the difference of potential between the two sides of the material. The mechanism of water evaporation electricity generation is different from the previous two types. The capillary seepage could be governed by water evaporation that generates a surface charge‐induced ionic effect in the charged channel of 2D materials and then forms a dynamic equilibrium ion concentration gradient in the flow direction. Further, the concentration gradient will cause the movement of ions and carriers, thereby generating voltage (Figure 2G–I).^[^ ^11^, ^20^, ^41^ ^]^

## 3 2D CARBON NANOSHEETS FOR HYDROVOLTAIC ELECTRICITY GENERATION

2D carbon materials show extraordinary sensitivity to adsorbed substances,^[^ ^4^ ^]^ so they are widely used in hydrovoltaic electricity generation. The most prominent 2D carbon nanomaterial is graphene, a unique 2D structure by sp^2^ hybridization of carbon atoms. As the thinnest 2D carbon materials,^[^ ^29^ ^]^ the graphene can attract each other and accumulate through weak van der Waals force.^[^ ^50^, ^51^ ^]^ Due to its unique structure, the accumulated graphene possesses the features of high electron mobility, superior mechanical properties, relatively high electron mobility, and large specific surface area.^[^ ^52^, ^53^, ^54^ ^]^ Moreover, graphene can be assembled into three‐dimensional (3D) nanostructures such as film,^[^ ^38^ ^]^ foam,^[^ ^28^ ^]^ sponges,^[^ ^27^ ^]^ could satisfactorily inherit excellent character from individual graphene. This is attributed to the constituent carbon atoms in these materials, mainly through van der Waals interactions.^[^ ^51^ ^]^ Besides, chemical methods can usually be used to process graphene to obtain oxygen‐containing functional groups and structural defects,^[^ ^55^, ^56^, ^57^ ^]^ including GO and rGO. Further research found that the functional groups on the surfaces of GO are sensitive to water, thus GO materials show rapid adsorption and desorption capacity of water molecules in humid surroundings.^[^ ^58^ ^]^ These can make graphene materials possess good performance in hydrovoltaic electricity generation.^[^ ^58^ ^]^

### 3.1 Moisture electricity generation

GO‐based materials are wildly used in moisture electricity generation. When the GO material is exposed to a moist environment, it can form the concentration gradient through certain treatments to generate the electrical output and internal electric field due to abundant functional groups.^[^ ^11^, ^29^ ^]^ Therefore, it is necessary to make a material that can establish a charge concentration gradient for facilitating charge migration. Usually, in moisture electricity generation, graphene is further assembled into 3D nanostructures, such as films and foams. Besides, some processing methods can be conducted to improve the material in these two forms to meet power generation requirements.

**FIGURE 3:**
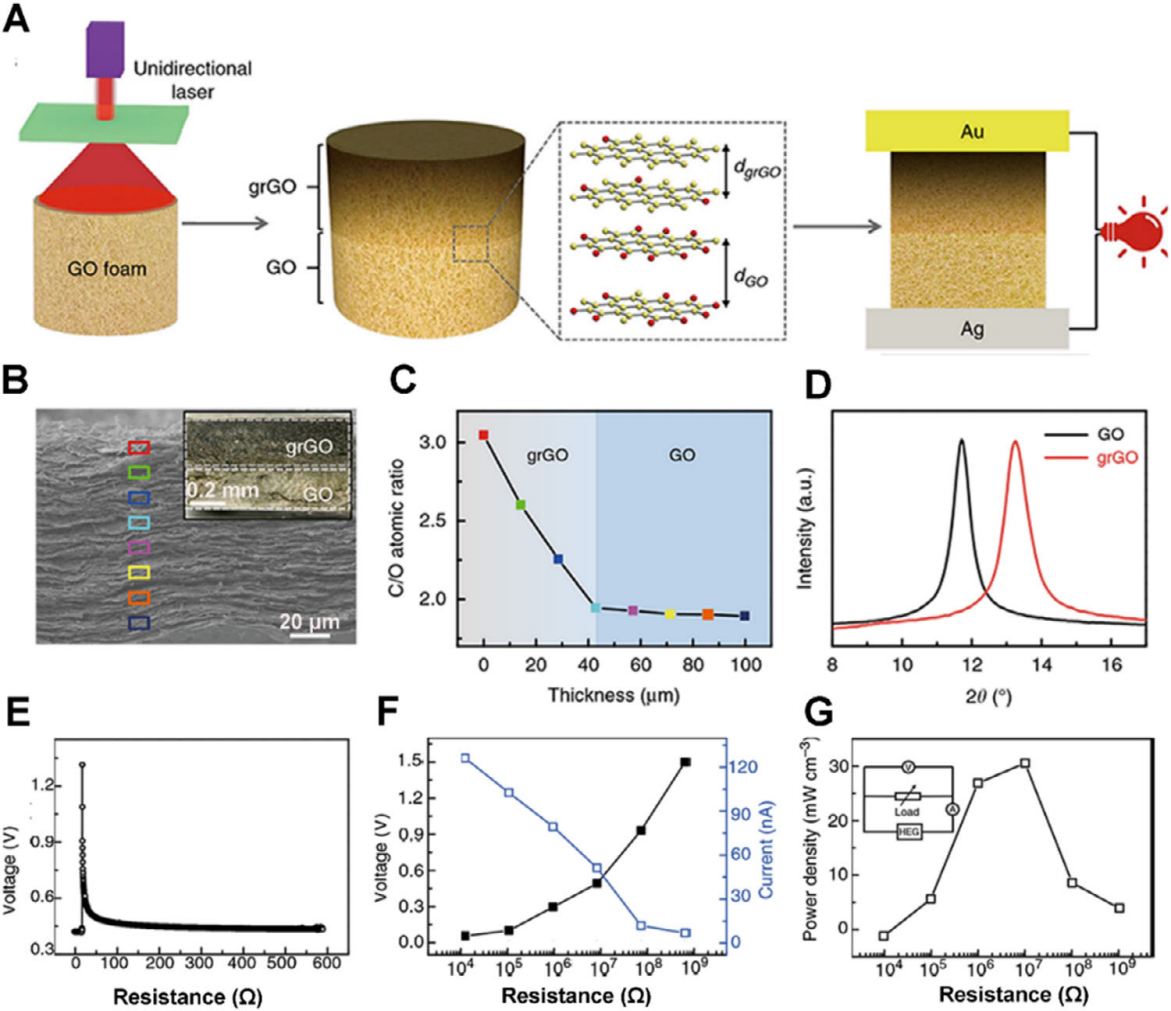
Graphene foam generates electricity in a humid environment. (A) The preparation process of GO foam. (B) Cross‐sectional scanning electron microscopy (SEM) image of GO foam. (C) The C/O ratio in the region of grGO and GO. (D) X‐ray diffraction pattern of the region of grGO and GO. (E) The voltage output of hydroelectric generators. (F,G) Output voltage, current, and power density of the hydroelectric generators with different electric resistors. Adapted with permission.^[^ ^28^ ^]^ Copyright 2018, Nature Publishing Group.

Graphene foam is of enough thickness to be modified with oxygen‐containing functional groups in a gradient via chemical methods for charge transfer. Qu's group reported a GO foam and then processed it through the thermal annealing method. The foam produces a large gradient of oxygen‐containing functional group concentration, generating a voltage of 0.2 V in a humid environment.^[^ ^36^ ^]^ Later, they further increased the thickness of the graphene layer, which can generate more movable ions to improve performance. In addition, the modification method of graphene has also been improved. Compared with thermal annealing treatment, laser treatment is more controllable. The laser intensity will decrease as the depth of the GO block increases, resulting in an oxygen‐containing functional group gradient (Figure 3A),^[^ ^28^ ^]^ and there will also be an unreduced GO layer (Figure 3B). As a result, the C/O of the top gradient rGO (grGO) layer was much higher than that of the bottom GO layer (Figure 3C,D). In order to further explore the application prospects of the hydrovoltaic electricity generator, an induced voltage was generated by absorbing moisture from the highly humid atmosphere (Figure 3E). At the same time, a short‐circuit current was generated. Under the resistance load of 10 MΩ, an optimal power density could reach to 32 mW cm^−3^ (Figure 3F,G). However, the voltage cannot be maintained at a high voltage for a long time because the desorption of water molecules will cause a voltage drop.

**FIGURE 4:**
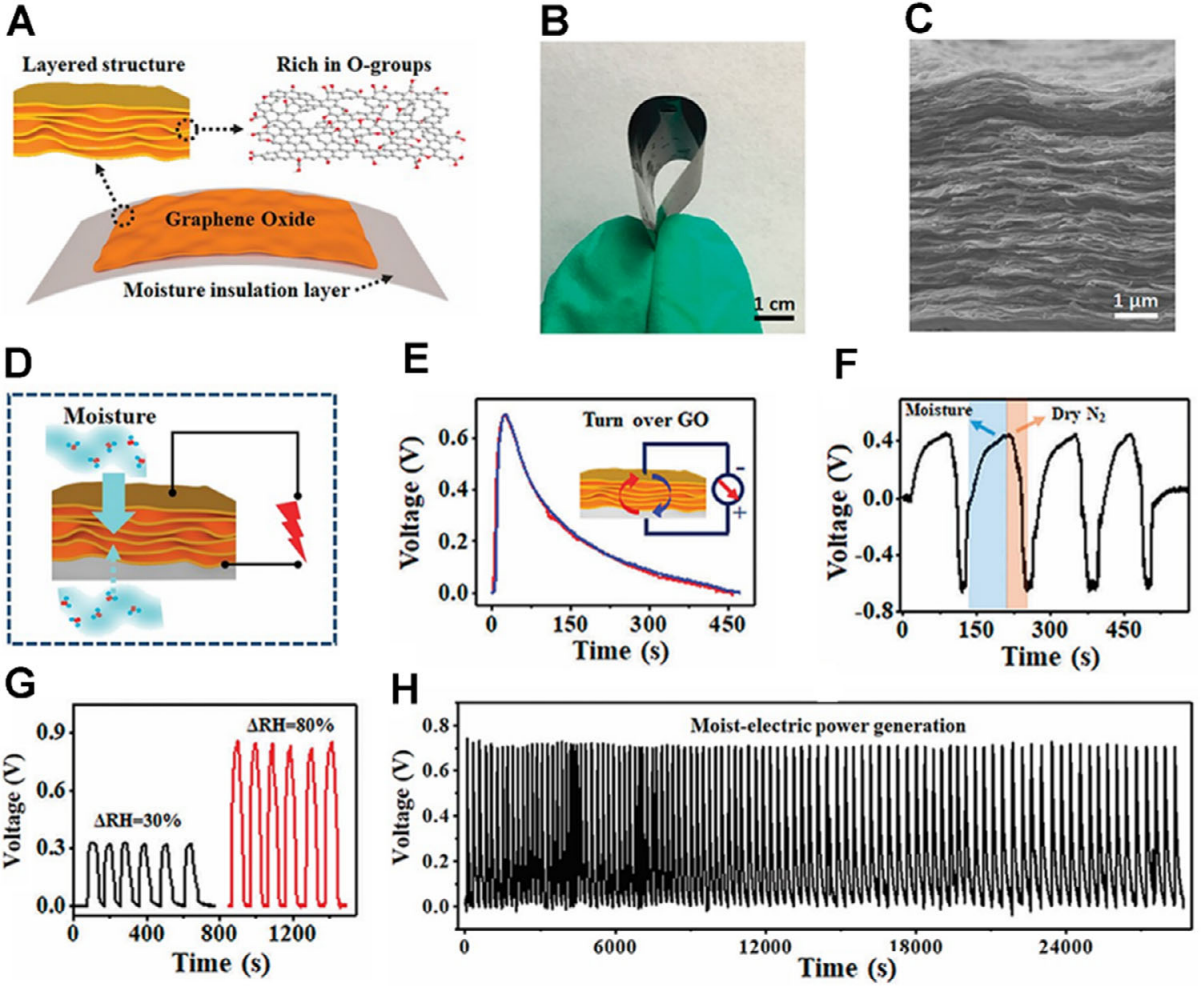
Graphene film generates electricity in a humid environment. (A) Schematic diagram of the generator. (B) The flexibility of the graphene oxide (GO) film. (C) GO film in a well‐ordered stacking morphology. (D) Schematic diagram showing moisture induced electricity generation. (E) The voltage before and after the GO/MIS bilayer flip. (F) The change of voltage by alternating N_2_ gas humidity. (G) The voltage of generator at different humidity. (H) Voltage output cycles of a generator. Adapted with permission.^[^ ^38^ ^]^ Copyright 2018, Royal Society of Chemistry.

Compared with graphene foam, due to the thinner thickness of graphene film, it is difficult to achieve a gradient in oxygen‐containing functional groups through the above processing methods. Qu et al. reported asymmetric moisture input to different material areas and applied this method to moisture electricity generation. They directly printed the GO onto a substrate that can insulate the environmental moisture, and named it as MIS (Figure 4A–C).^[^ ^38^ ^]^ In this way, moisture can only enter from one end near the bare side, thereby controlling the direction of ion flow (Figure 4D). To reveal the significance of asymmetric moisturizing, follow‐up experiments were carried out. First, the GO film was turned over on the MIS, and the voltage output was reproduced, eliminating the influence of the special nanostructures and oxygen‐containing functional group gradients of GO films (Figure 4E). Next, alternating wet and dry N_2_ flow alternately to the sample will cause periodic relative humidity (RH) changes, which results in alternating positive and negative voltage cycles (Figure 4F). This result further confirms the importance of the moisture environment in moisture electricity generation. As shown in Figure 4G, the voltage produced by the device can be up to 0.9 V at a RH variation of 80%.^[^ ^38^ ^]^ The device could maintain relatively high voltages for a long time by repeating the hydration and dehydration processes (Figure 4H).

### 3.2 Waterdrop electricity generation

In addition to moisture electricity generation, water drop electricity generation is another efficient way of harvesting hydrovoltaic energy using graphene materials. Classical electrokinetic theory points out that the liquid flowing along the charged surface of a thin‐film electrode can create the streaming potential.^[^ ^59^, ^60^, ^61^, ^62^ ^]^ When the liquid stops moving, the induced voltage will zero. Similarly, the opposite voltage will generate while the liquid moves reverse. Besides, the generated voltage magnitude can be affected by the speed and quantity of the liquid flow.^[^ ^16^, ^28^, ^63^ ^]^

**FIGURE 5:**
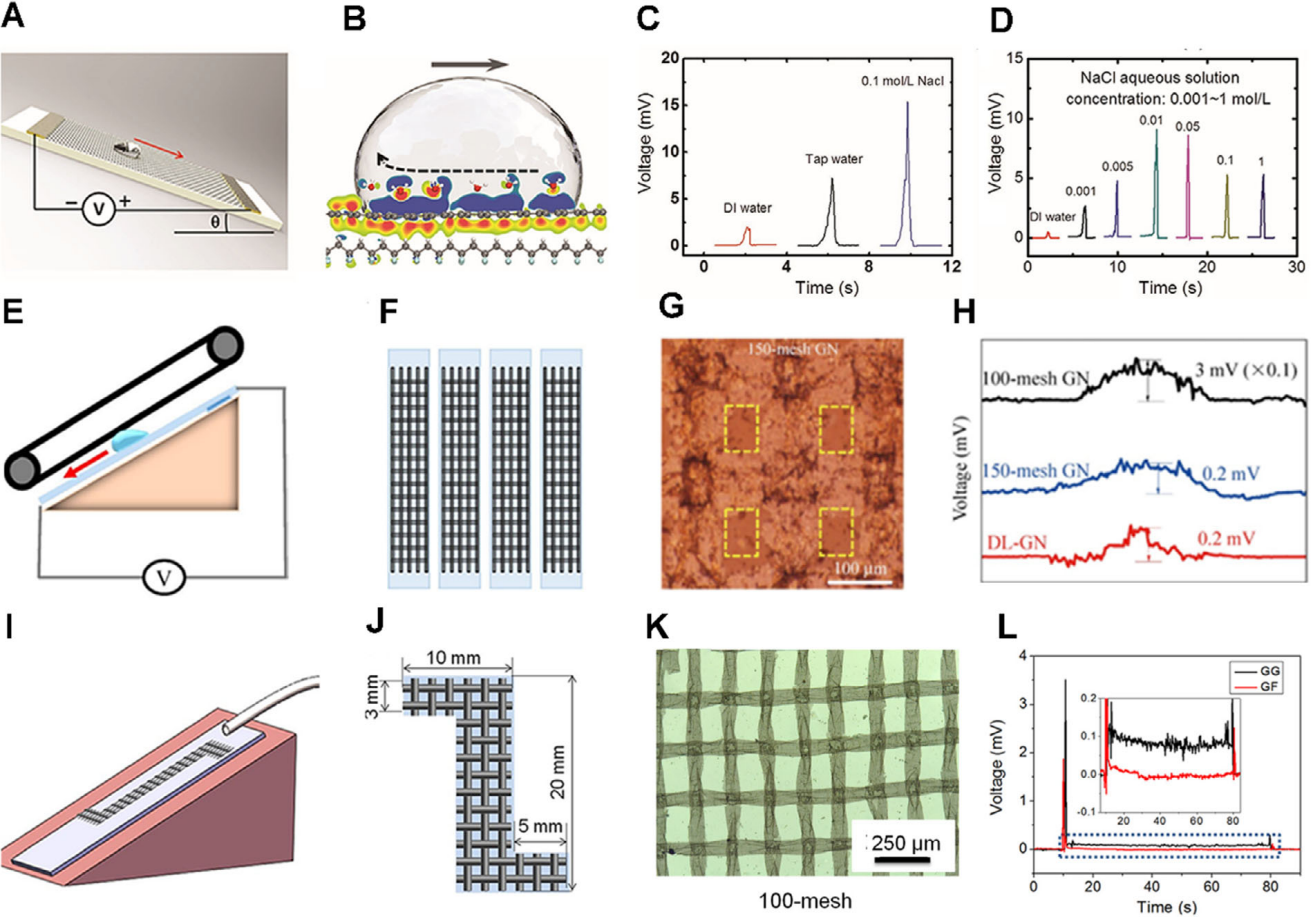
Waterdrop electricity generation on graphene‐based materials. (A) The diagrammatic drawing of experimental setup for power generation based on graphene‐PVDF film. (B) Change of charge density at the interfacial reaction by a moving pure water droplet . (C,D) The induced voltage generated by different solutions and diverse concentrations of NaCl. Adapted with permission.^[^ ^49^ ^]^ Copyright 2017, Wiley‐VCH. (E,F) Experimental setup for generating electricity based on graphene networks. (G) Optical photographs of 150‐mesh graphene networks. (H) The voltage of GN with different mush numbers induced by the flowing of NaCl droplets. Adapted with permission.^[^ ^64^ ^]^ Copyright 2015, Springer. (I,J) Experimental setup for electricity generation based on graphene girds. (K) Optical photographs of 100‐mesh graphene girds. (L) The graphene grid and graphene film produce a contrast of voltage. Adapted with permission.^[^ ^65^ ^]^ Copyright 2015, AIP Publishing.

Inspired by the above phenomenon, Zhong and co‐workers transferred monolayer graphene strips onto the polyvinylidene fluoride (PVDF) film to design a nanogenerator (Figure 5A).^[^ ^49^ ^]^ The droplet passes by the graphene surfaces in this nanogenerator can produce a millivolt voltage, attributed to the charge density change at the water–graphene–PVDF interface (Figure 5B). Thus, the charge transfer at the interface of solid and liquid is the key to electricity generation. Moreover, the different quantity of liquid affects the flow‐induced voltage output. Deionized (DI) water, tap water, and different concentrations of NaCl solutions were used for comparison. Consequently, the water droplets with higher conductivities could result in higher flow‐induced voltage output (Figure 5C,D). Besides, according to the mechanism of water drop electricity generation, the dynamic charge transfer processes between water and the surfaces of substrates are crucial, thus the influences of substrates could not be neglected. With the DI water flow, the monolayer graphene with three different substrates responds different induced voltage. There is a possible reason that the kind of charged ions attracted and the density of the EDL are mainly determined by the substrates, but the mechanism behind this phenomenon is still unclear.

Compared with graphene flakes, the graphene network (GN) has a flexible structure and high mechanical strength and can generate a greater voltage in response to the movement of ionic liquid droplets. The new interconnected GN could be fabricated on copper meshes by chemical vapor deposition (Figure 5E,F).^[^ ^64^ ^]^ The mesh in the structure has a great influence on electricity generation. The mesh in the structure will cause bumps on the GN surface (Figure 5G), enhancing electricity generation and increasing the utilization of ions. However, too many meshes will cause the hole to get deeper and affect ion transfer, resulting in decreased voltages (Figure 5H).

Different from the above reports that voltage generation requires droplets flowing alone on the surfaces of nanomaterials, Zhu's group designed a graphene‐based grid (GG) structure,^[^ ^65^ ^]^ which could generate voltage when the liquid moves continuously on the surfaces. The GG comprises numerous copper woven mesh with interlacing and roughness characteristics (Figure 5I,J). Besides, the mesh numbers could also affect the electrical output for GG, and the highest voltage was obtained with a 100‐mesh GG (Figure 5K). For the GG, a peak of 3.5 mV could be obtained when the liquid began to flow on the surface constantly. After the liquid filled the flow channel of the GG, the voltage declined to only 0.1 mV. Nevertheless, GG could remain stable when the liquid flowed smoothly. In contrast, the monolayer graphene film only generated voltage at the onset and end of the flow under the same conditions (Figure 5L).

### 3.3 Water evaporation electricity generation

Compared with moisture and water drop, water evaporation has its unique advantage of occurring anywhere and anytime, regardless of weather and environmental conditions. Inspired by this transpiration phenomenon, we can construct capillary channels in the 2D carbon nanomaterial structure to realize water evaporation to induce capillary transport.^[^ ^14^, ^24^, ^66^, ^67^ ^]^ In this process, the interaction between water molecules and the surfaces of materials can also be used to generate electrical energy output. The previous two ways of hydrovoltaic electricity generation can produce power only at certain conditions, such as a stable humid environment and external hydraulic flow.^[^ ^27^ ^]^ These limiting conditions hinder the development and potential application of hydrovoltaic energy. By contrast, water evaporation electricity generation is less affected by those restrictions. Because it depends on natural water evaporation, which is a ubiquitous phenomenon all around us, it is therefore theoretically possible to turn into the neoteric self‐powered devices.

**FIGURE 6:**
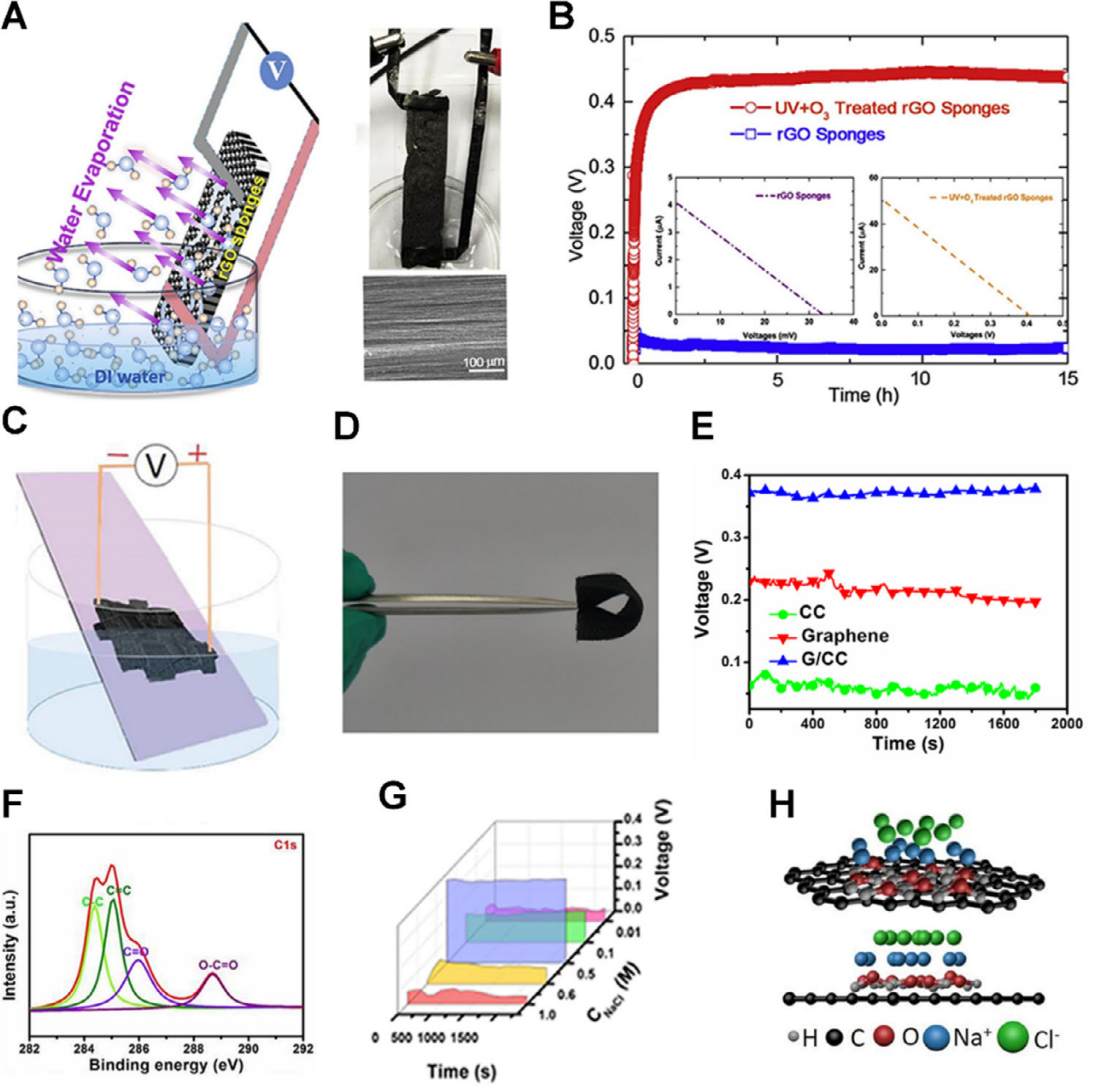
Electricity generation induced by water evaporation over graphene‐based materials. (A) Electricity generation induced by water evaporation over reduced graphene oxide (rGO) sponges. (B) Comparison of the performance of rGO sponges after plasma treatment and rGO sponges without plasma treatment. Adapted with permission.^[^ ^27^ ^]^ Copyright 2019, Elsevier. (C) Schematic diagram of evaporation induction point based on carbon cloth/graphene (G/CC). (D) The photograph of the G/CC. (E) Comparison of different carbon material properties. (F) X‐ray photoelectron spectroscopy spectra and fitting curves of G/CC. (G) Schematic diagram of the performance of different concentrations of NaCl solution. (H) The principal diagram of generating electricity over G/CC in NaCl solution. Adapted with permission.^[^ ^34^ ^]^ Copyright 2018, Elsevier.

The application of carbon material in water evaporation‐induced electricity generation has been recently reported. For example, a CB sheet was grown onto a quartz strip by a simple ethanol flame method. Subsequently, the CB suffered air plasma treatment, increasing the concentration of oxygen‐bearing functional groups and strengthening the interaction with the water–CB interface. As a result, the short circuit current and open‐circuit voltage were measured to be 100 nA and 1 V, respectively.^[^ ^5^ ^]^ Yao et al. explored the application of graphene in water evaporation electricity generation.^[^ ^27^ ^]^ A graphene sponge was fabricated by a self‐assembly method followed by a post‐annealing and plasma treatment. The graphene sponge has a porous structure and oxygen‐containing functional groups on the surface, generating a stable voltage under natural water evaporation (Figure 6A). As shown in Figure 6B, the voltage generated by the plasma‐treated rGO could keep a relatively stable voltage output of ∼0.44 V, while the untreated rGO could not generate a voltage at the same condition. This result confirms that the oxygen‐containing functional groups are key to gaining higher output power in water evaporation electricity generation.

Graphene‐based composite materials have attracted huge attention in water evaporation‐induced electricity generation. Wang's group used electrophoretic deposition to attach graphene oxide to carbon cloth (CC).^[^ ^34^ ^]^ Carbon cloth/graphene (G/CC) could be fabricated into a pliable and portable device for water evaporation electricity generation (Figure 6C,D). Compared with CC and graphene, G/CC can generate higher voltage under the same condition (Figure 6E). This should be attributed to more oxygen‐containing functional groups on the G/CC surfaces (Figure 6F). Further, when G/CC is immersed in NaCl solution, the overlapped EDL would be formed at the solid‐liquid interfaces.^[^ ^14^, ^68^, ^69^ ^]^ As the evaporation of the NaCl solution, the ions in the EDL will migrate to generate voltage output. The experiment shows that solutions of different concentrations will produce a voltage of different magnitudes (Figure 6G). Wang et al. proposed an explanation for this phenomenon. Owing to the increase of the solution concentration and negative surface charge of graphene, more positive ions transfer to the surfaces of materials and constantly accumulate. Because of its negative adsorption energy, the negative ions are excluded. The excessive positive ions will migrate directionally and redistribute, and a voltage will generate during these processes.^[^ ^16^ ^]^ However, since the migration of ions is an endothermic process, the output voltage will decrease as the solution concentration increases (Figure 6H).

## 4 2D TRANSITION METAL OXIDES AND SULFIDES FOR HYDROSTATIC ELECTRICITY GENERATION

Some 2D oxide and sulfide nanomaterials have also been applied to hydrovoltaic electricity generation, such as water wave, water drop, water evaporation, and so on. These materials can form capillary channels and have a high zeta potential on their surfaces, which is propitious for energy harvesting. When water molecules pass through narrow channels with a charged surface, the potential and current are generated with the counterions in the EDL moving along with the water molecules.

**FIGURE 7:**
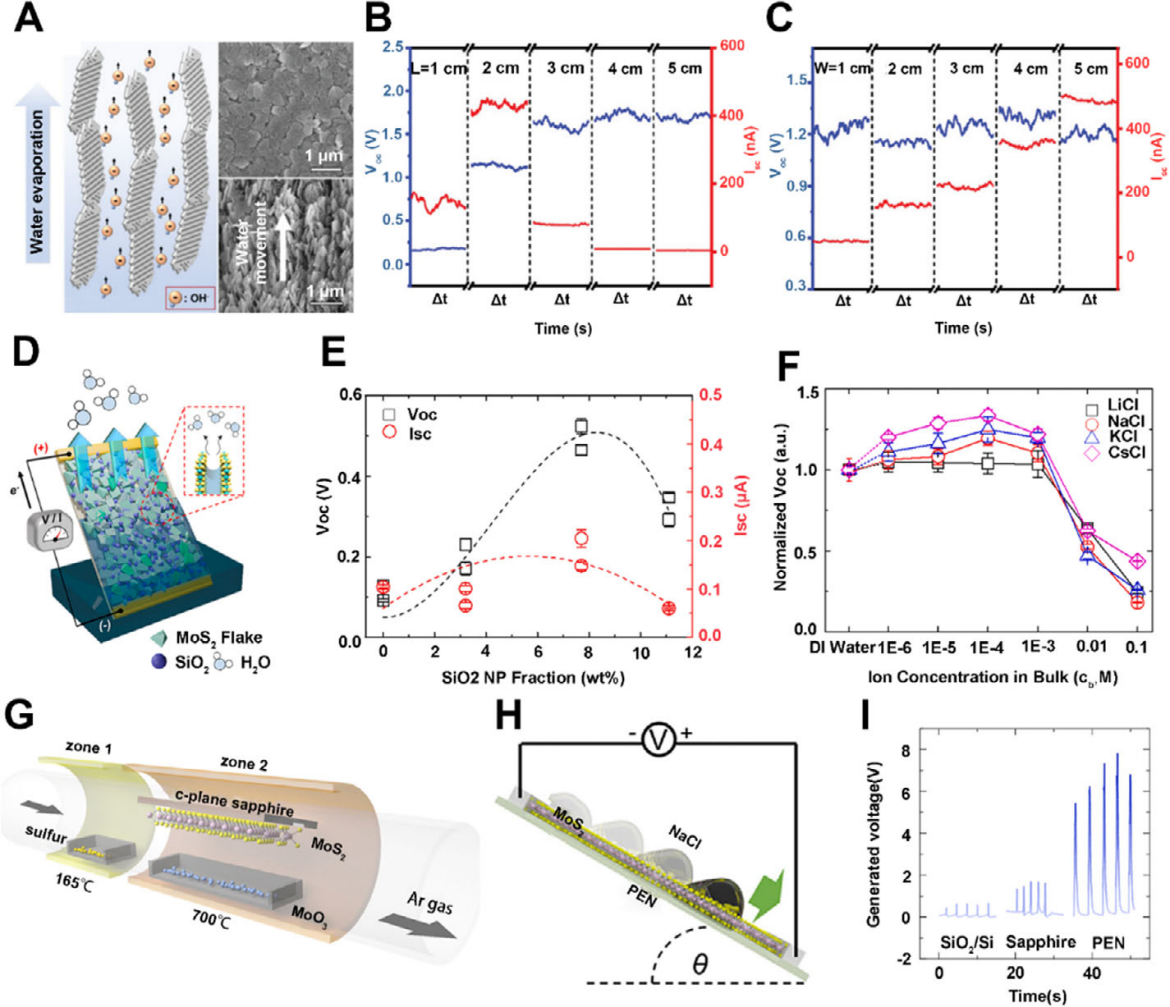
Hydrostatic electricity generation over 2D transition metal oxides and sulfides. (A) Schematic illustration of the generating electricity from water evaporation and SEM images of the AlOOH/UIO‐66 film. (B) Performance of devices with different lengths. (C) Performance of devices with different widths. Adapted with permission.^[^ ^45^ ^]^ Copyright 2020, Wiley‐VCH. (D) Schematic illustration of electricity generation by water evaporation in MoS_2_/SiO_2_ composite film. (E) *V*_oc_ and *I*_sc_ is a function of a mass fraction of SiO_2_ NP. (F) Normalized *V*_oc_ in different kinds and concentrations solution. Adapted with permission.^[^ ^43^ ^]^ Copyright 2021, Elsevier. (G) Schematic illustration of preparation method of MoS_2_ film. (H) Schematic diagram of ionic liquid droplets slid on the MoS_2_ film surface to generate electricity. (I) Effect of different substrates on voltage when liquid moves on MoS_2_ surface. Adapted with permission.^[^ ^44^ ^]^ Copyright 2020, Elsevier.

Zhang et al. created a hybrid nanomaterial consisting of UIO‐66 nanoparticles coated on AlOOH nanoflakes (AlOOH/UIO‐66) (Figure 7A).^[^ ^45^ ^]^ The devices made of AlOOH/UIO‐66 hybrid films were investigated to obtain electricity from water evaporation, which could achieve an average open‐circuit voltage of ∼1.63 V. In addition, the dimensions of the AlOOH/UIO‐66 hybrid film could affect the generated voltages and currents of the devices. While keeping the width constant, the voltage gradually increased to the maximum level with the increase in length (Figure 7B). Interestingly, the change in width did not influence the voltage but influenced the current. As shown in Figure 7C, the current increased gradually with the increase in width, while keeping the length unchanged. This phenomenon could be explained that the voltage and current output of the devices can be improved by connecting several devices in series or in parallel, which is similar to a commercial battery.

Similarly, sulfide nanomaterial can also be applied to harvest energy from water evaporation. Kim's group reported a composite film fabricated by mixing molybdenum disulfide (MoS_2_) and silica nanoparticle (SiO_2_ NP) with a post‐annealing treatment (Figure 7D).^[^ ^43^ ^]^ The hydrophilicity and resistance of the composite film can be controlled by adjusting the content of charged NPs in the MoS_2_ matrix. The outputs of voltage and current first increased and then decreased with the increase of SiO_2_ NP fraction (Figure 7E). This phenomenon could be explained by the varying interactions between the charge carriers of MoS_2_ and the counterions in the nanocapillarity. The dependency of voltage and current also exhibited a similar tendency in different alkali ion solutions, which showed the difference of ion species‐dependency from the conventional streaming current (Figure 7F). Moreover, MoS_2_ can generate electricity through the dynamic liquid drop. Through chemical vapor deposition, large‐area single‐layer MoS_2_ films were synthesized as a nanogenerator.^[^ ^44^ ^]^ They employed a surface energy‐assisted transfer method to transfer the MoS_2_ onto polyethylene naphthalene (PEN) substrates using polystyrene supporting films (Figure 7G).^[^ ^70^, ^71^ ^]^ When the ionic liquid droplets slid smoothly on the single‐layer MoS_2_ film surface (Figure 7H), an excellent output voltage of 4 – 6 V was instantaneously observed, which is much higher than that of multi‐layer MoS_2_ film (∼70 μV).^[^ ^72^ ^]^ Besides, the output voltages of MoS_2_ nanogenerator on various substrates were different, with the MoS_2_/PEN device showing the largest generated open‐circuit voltage among all studied devices (Figure 7I). The possible reason could be ascribed to the enhanced sheet resistance and the improved absorption capability of Na^+^ ions by the MoS_2_ layer on PEN substrate, leading to improved electricity generation capacity of the MoS_2_ nanogenerator.

## 5 2D LDH FOR HYDROSTATIC ELECTRICITY GENERATION

LDH can generate a continuous electrical output by the evaporation of natural water. LDH is a type of ionic lamellar compound consisting of positively charged layers with an interlayer area comprising charge anions and solvation molecules. Due to the high charge density and anion concentration of interlamination, LDH also shows extraordinary sensitivity to adsorbed substances, which can be widely used in hydrovoltaic electricity generation.^[^ ^73^ ^]^ Compared with other 2D materials, LDH possesses the features of intrinsic high surface positive charge density, inherent hydrophilicity, and the internal nanochannels or nanopores between LDH flakes.^[^ ^73^, ^74^, ^75^, ^76^ ^]^ These advantages contribute to the excellent natural water evaporation electricity generation performance of LDH.

**FIGURE 8:**
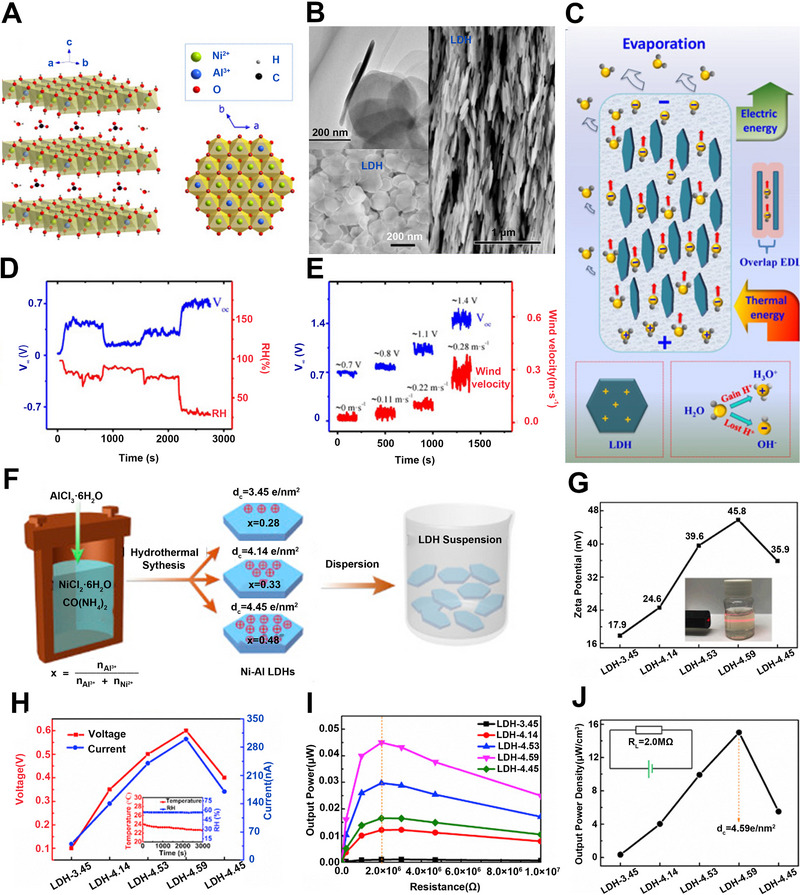
Hydrostatic electricity generation over 2D layered double hydroxides (LDHs). (A) Schematic illustration of the structure of Ni‐Al LDH. (B) Cross‐sectional SEM and TEM image of the Ni‐Al LDH film. (C) Schematic diagram of Ni‐Al LDH for water evaporation electricity generation on a microscopic level. (D) Response of *V*_oc_ to the cyclic variation of different RH. (E) Response of *V*_oc_ to the cyclic variation of wind velocity. Adapted with permission.^[^ ^41^ ^]^ Copyright 2019, Elsevier. (F) The fabrication of the Ni‐Al LDH with different *d*_c_. (G) The zeta potentials of different Ni‐Al LDH colloidal solutions. (H) Relatively stable *V*_oc_ and *I*_sc_ of five generators under laboratory conditions. (I) External *R*_L_ function of five generators output power. (J) *d*_c_ function of output power density under the *R*_L_ of 2.0 MΩ. Adapted with permission.^[^ ^40^ ^]^ Copyright 2020, Elsevier.

Ni‐Al LDH was reported to be directly utilized in electricity generation without further regulation.^[^ ^41^ ^]^ It has a layered structure (Figure 8A), which favors the directional flow of ions between layers. The Ni‐Al LDH can be painted on the substrates to ensure its layered structure, which is stacked layer‐by‐layer in an orderly manner, thus directly outputting power by water evaporation. (Figure 8B). Based on the above discussion, the migration of hydroxyl ions in layered structures is a crucial part of the process of electricity generation. Therefore, it is important to explore an approach to improve the transport behavior of ions. Recently, Sun's group reported that the migration of ions can be enhanced by several orders of magnitude by intensively overlapping EDL (Figure 8C). They analyzed the effects of humidity and wind velocity on the performance to further explore the performance of the Ni‐Al LDH generator. As a result, the RH and wind velocities are the key external factors that affect the voltage of LDH in reverse and positive directions, respectively. (Figure 8D,E).

Compared with external factors, internal factors affect the performance of LDH generators more directly. The electricity generation performance induced by natural water evaporation could be improved by regulating the surface charge density of the LDH.^[^ ^40^ ^]^ Tan and co‐workers fabricated Ni‐Al LDH powders with the different molar ratios of Al^3+^ to Ni^2+^ via a hydrothermal method (Figure 8F).^[^ ^77^ ^]^ The higher surface charge density resulted in a larger surface potential, which could enhance water–LDH interaction. Among all studied samples, the LDH sample with the highest charge density of 4.59 e/nm^2^ showed the highest zeta potential (Figure 8G). When applied in natural water evaporation, this sample delivered a high voltage of ∼0.6 V and a large current of ∼310 nA (Figure 8H). Consequently, the highest output power density of ∼15 μW/cm^3^ could be achieved when the external resistance was ∼2.0 MΩ (Figure 8I,J).

## 6 DEVICES OF THE HYDROSTATIC ELECTRICITY GENERATION

2D materials have enormous potential in practical devices because the output power and the energy conversion efficiency are continually increasing.^[^ ^78^, ^79^, ^80^ ^]^ As for hydrovoltaic electricity generation, many studies show that devices fabricated with 2D material are viable to generate electricity. Among them, devices can be divided into flexible and non‐flexible devices. At present, non‐flexible devices made of AlOOH and other 2D materials have great output power. However, compared with non‐flexible device, flexible devices can adapt to different environments while maintaining excellent output power because of their own properties. Therefore, flexible self‐powered devices fabricated with 2D materials are promising for developing sustainable nodes in the future applications, including sensor and external power supply.

**FIGURE 9:**
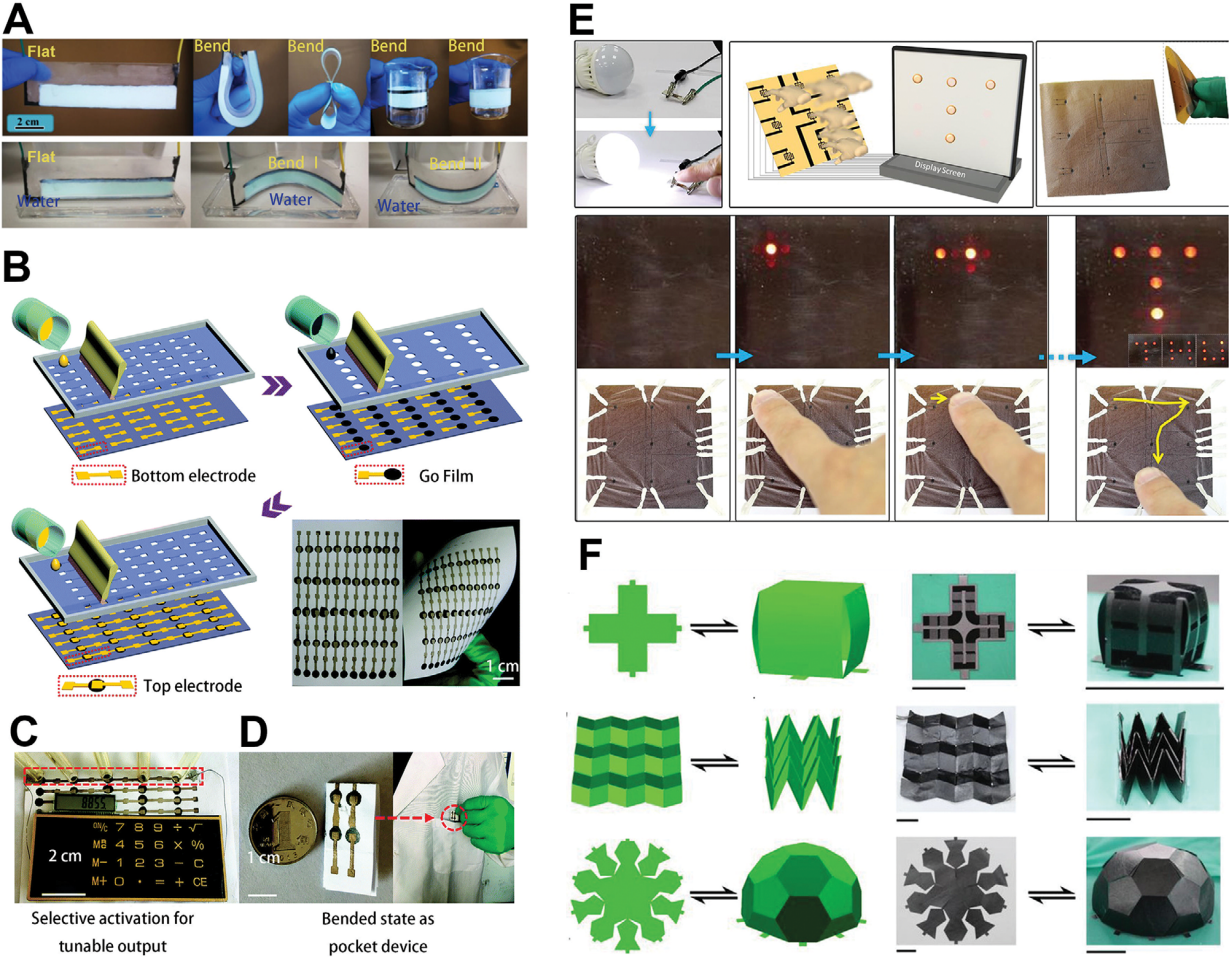
Flexible devices of hydrostatic electricity generation fabricated by 2D materials. (A) Ni‐Al layered double hydroxide flexible generator by bending to various degrees under dry and saturated circumstances. Adapted with permission.^[^ ^41^ ^]^ Copyright 2019, Elsevier. (B) Screen printing of large‐scale device fabrication of graphene oxide (GO) film on flexible paper sheets. (C) GO device power the calculator. (D) Portable performance of the device. Adapted with permission.^[^ ^38^ ^]^ Copyright 2018, Royal Society of Chemistry. (E) A touchless moisture power‐generating device. Adapted with permission.^[^ ^35^ ^]^ Copyright 2017, Elsevier. (F) GO film power devices with different spatial shapes: cubic box, Miura‐ori, and half‐football structure. Adapted with permission.^[^ ^81^ ^]^ Copyright 2019, Wiley‐VCH.

With the development of water evaporation electricity generation, various 2D materials have been used in this field, such as LDH, MoS_2,_ and AlOOH. The Ni‐Al LDH painted on substrates showed high flexibility. The as‐prepared device can bear large‐scale bending deformation, and still shows the well‐preserved performance as the unbending device in the performance test of electricity generation (Figure 9A).^[^ ^41^ ^]^ Compared to water evaporation electricity generation, moisture electricity generation greatly improves the portability of the devices. Through a simple screen‐printing method, GO was uniformly printed on a moisture‐proof printing plate, resulting in a large‐scale GO film on flexible paper sheets (Figure 9B).^[^ ^38^ ^]^ It is found that the output is enough to power an electrical calculator by simply connecting many GO film generators (Figure 9C). Meanwhile, the as‐prepared flexible paper generators can be put into a pocket after folding, showing high portability (Figure 9D). Apart from the screen‐printing, laser fabrication of rGO is another method to fabricate the flexible device.^[^ ^35^ ^]^ The as‐fabricated rGO flexible device can generate electrical energy by absorbing ambient water molecules (Figure 9E). Besides, the flexible device can be bent arbitrarily without any significant performance loss—even the strain changes from 100% to 2000%.^[^ ^81^ ^]^ As a result, one can design the devices in cubic boxes, pyramids, football, and more complicated shapes. In the various forms, the voltage of the device was stably held between 1.05 and 1.27 V, which is promising for application in complex conditions (Figure 9F).

**FIGURE 10:**
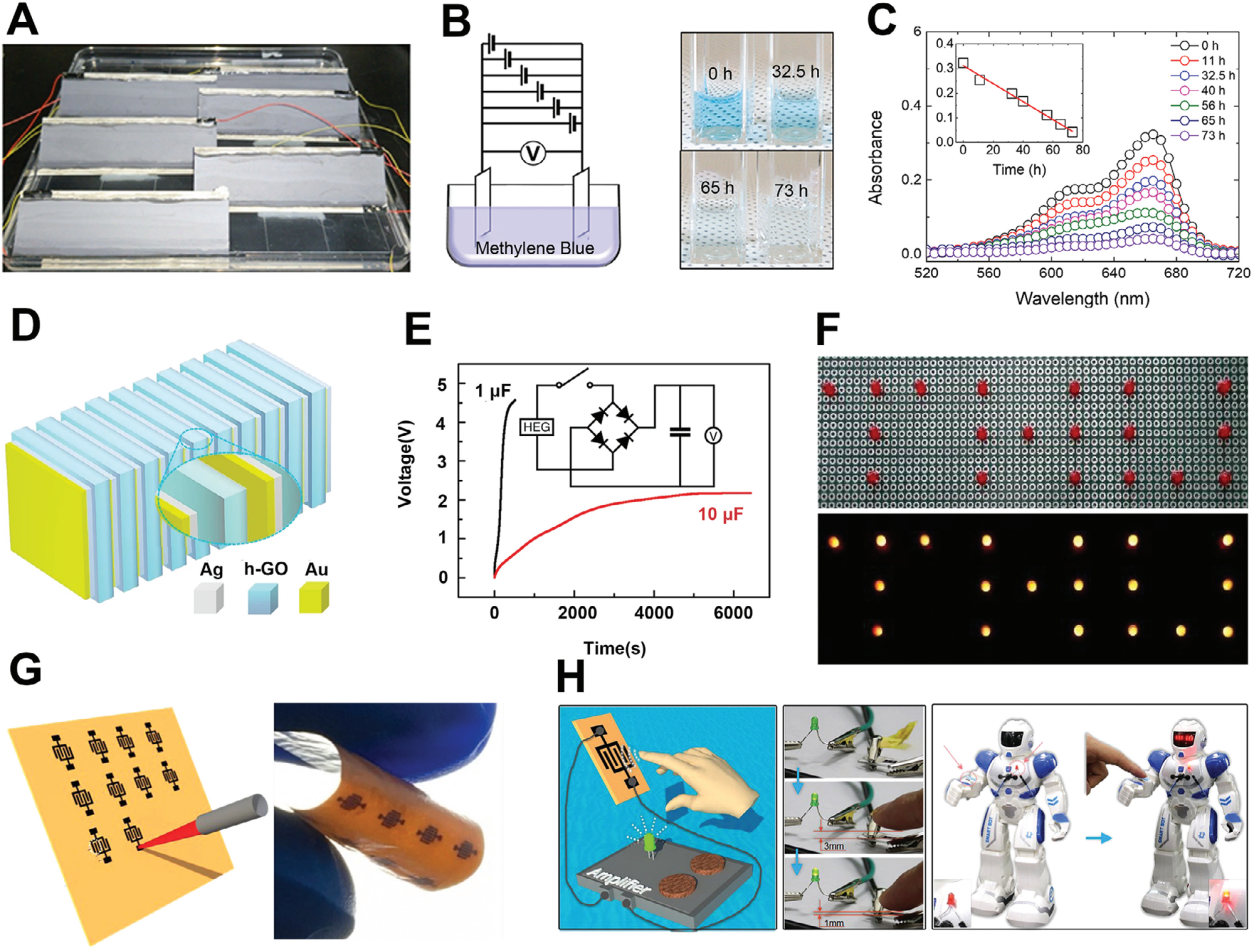
Applications of hydrostatic electricity generation devices fabricated by 2D materials. (A) Physical drawing of water evaporation generator. (B) The device applied to decompose MB solution. Left is schematic experimental setup and the right is corresponding decoloration images. (C) The absorbance of MB solution as a function of wavelength. Adapted with permission.^[^ ^43^ ^]^ Copyright 2021, Elsevier. (D) Schematic of an assembled hydroelectric generator. (E) Voltage‐time curves when the device charges the capacitor. The inset shows the charge storage circuit. (F) Various electronics powered by the stored charge in 1 μF capacitor. Adapted with permission.^[^ ^28^ ^]^ Copyright 2018, Nature Publishing Group. (G) Schematic of flexible GO device for laser processing. (H) The device applied in moisture sensor. Schematic diagram of a finger position variation (left), the LED brightness changing with the finger position variation (middle), and the device attached top gradient rGO to the robot as a sensor (right). Adapted with permission.^[^ ^35^ ^]^ Copyright 2017, Elsevier.

Beyond these flexible devices, the ability of self‐powered devices for practical applications can help us explore hydrovoltaic electricity generation constantly. Six‐parallel connected devices of water evaporation‐induced electricity generation could produce a high voltage, which is sufficient for electrochemical decomposition of methylene blue (MB) solution, when applied as a power supply in an electrochemical system (Figure 10A,B).^[^ ^43^ ^]^ The decolorization of the MB solution was noticeable, and a complete decolorization was almost achieved within 73 h (Figure 10C). Compared to parallel connections, the hydroelectric moisture generators can directly obtain a high voltage by an efficient stacking method (Figure 10D).^[^ ^28^ ^]^ Each power unit with a layer‐by‐layer stacking strategy can absorb moisture from the environment and charge the capacitor (Figure 10E). The fully charged capacitors are sufficient to power some electronic devices, such as LED and nixie tube arrays (Figure 10F). As for the hydroelectric moisture generators, any variation in ambient humidity will lead to changes in their electrical output. This makes hydroelectric moisture devices have a capability of the application as a self‐powered sensor. Cheng et al. used direct laser writing method to pattern pairs of conductive rGO microelectrodes on GO film to fabricate the moist electricity generation device (Figure 10G).^[^ ^35^ ^]^ The appreciable electric output can be generated with the variation of moisture. Based on this, it can perceive the moisture on the approaching fingers through the changes of induced voltages (Figure 10H). Therefore, the touchless sensing devices may provide a new pathway on touchless switches and even the alert reminding people of far away.

## 7 CONCLUSION AND PERSPECTIVE

Hydrovoltaic energy can convert thermal energy from the surroundings to electric power through spontaneous water evaporation, rendering it an emerging green energy technology with special superiority over other energy conversion technologies. This review summarizes the recent developments and applications of 2D materials in hydrovoltaic devices, including water evaporation electricity generation, moisture electricity generation, water drop electricity generation (Table 1). Recently, it has been exciting that optimized hydrovoltaic devices could deliver high output voltages by taking advantage of the effective transportation of ions in the nanochannels of 2D materials.

**TABLE 1:** Summary and comparison of hydrovoltaic energy

	Materials	Solution	Flow type	Potential (mV)	Current (μA)	Electricity output	Ref.
2D materials	Graphene oxide (GO) film	Water	Moisture	20	5	4.2 mW cm^−2^	[8]
	GO film	Water	Moisture	700	0.3	0.27 W m^−2^	[38]
	GO	Water	Moisture	600	∼1		[37]
	Reduced graphene oxide	Water	Moisture	1500	98–136		[28]
	GO film	Water	Moisture	70		12 mA cm^−2^	[35]
	GO	Water	Moisture	180	1.2		[81]
	GO film	Water	Moisture	400–700	2–25		[15]
	MoS_2_	NaCl	Water drop	5000	∼5		[44]
	Graphene‐piezoelectric	Water	Water flow	100			[49]
	AlOOH/UIO‐66	Water	Water evaporation	∼1630		6.5 μW cm^−3^	[45]
	MoS_2_/SiO_2_	Water	Water evaporation	∼800	0.25–0.3		[43]
	Layered double hydroxide (LDH)	Water	Water evaporation	700	1.3	16.1 μW cm^−3^	[41]
	LDH	Water	Water evaporation	600	∼0.3	15 μW cm^−3^	[40]
	Monolayer graphene	CuCl_2_	Water drop	∼30	∼1.7		[16]
Non‐2D materials	Carbon black	Water	Water flow	1000	0.15		[24]
	Carbon film	Water	Evaporation	1000	0.6		[11]
	Polyelectrolyte membrane	Water	Moisture	800	100		[57]
	TiO_2_	Water	Moisture	∼500		4 μW m^−2^	[12]
	Al_2_O_3_	Water	Water evaporation	∼2500	0.8	7.8 nW m^−2^	[82]

Although this field has achieved many remarkable progresses, some challenges for 2D materials in hydrovoltaic devices should be concerned. First, the mechanisms proposed so far need to be consummated. Although some mechanisms for hydrovoltaic electricity generation on nanomaterials have been proposed, they cannot clearly explain the electricity generation processes on 2D material. In situ technologies have a significant potential role in further understanding the interaction mechanism between solid materials and water. They can be able to study various physical and chemical processes at the solid/liquid interface in a static or dynamic state, which is a good tool to investigate the mechanism of hydrovoltaic electricity generation. In addition, advanced computational theories and simulation methods that calculate water–solid system at the quantum mechanical level are vital for obtaining deeper understanding of these mechanisms. Through these theories and methods, we can predict the physical or chemical properties of functional materials when in contact with water to the extent possible, and the corresponding dynamic processes and behavior during hydrovoltaic device operation. As good platforms for studying interactions between water and surfaces of nanomaterials at an atomic scale, 2D materials stand out from other nanomaterials. Future studies should focus on the adsorption/desorption of water molecules/ions on surfaces of 2D materials. The detailed dynamics between water molecules/ions in liquid and the carriers in 2D materials should also be investigated.

Second, the output performances reported were still far from practical applications. Although the voltages of hydrovoltaic electricity generation devices have reached the volt level, the generated currents are still too low, leading to low output power densities. The main reason should be the weak interaction between the water molecules and material surfaces. These performances cannot yet be comparable to those from traditional devices. The power needs to be further improved to a daily usable level. The 2D materials with nanosized pores or charged channels that reinforce the interfacial interaction, are key to obtaining relatively high output power in hydrovoltaic electricity generation. However, the research of 2D materials in this field is still at an early stage. A summary of the advantages and disadvantages of some 2D materials for hydrovoltaic electricity generation is listed in Table 2. It is apparent that there are still a lot of undiscovered materials which could also be used for construction of future hydrovoltaic devices, such as 2D transition metal carbides (MXenes) and 2D metal‐organic framework materials (MOFs). MXenes have tremendous characteristics, such as high surface areas and ease of functionalization, which could effectively enhance the interactions between them and water. MOFs are another emerging material with the advantage of high porosity and relatively large surface area. The interactions between the surfaces of MOF and water molecule can be accurately adjusted by changing the crystal structures and the morphology of MOF crystals. In addition, 2D heterostructures should be also studied in hydrovoltaic electricity generation. Due to combination of advantages of different 2D materials and other nanomaterials, the power output of devices fabricated by 2D heterostructures may increase dramatically. There have been some previous studies on two‐dimensional heterostructures about hydrovoltaic electricity generation, such as composite materials of this type by MoS_2_ and SiO_2_ NP.^[^ ^43^ ^]^ This indicates that heterostructures may be very efficient to develop hydrovoltaic nanogenerators that can convert different low‐grade energy from the environment. In the future, the studies should pay attention to explore ways to increase or adjust the micro‐nano structure of 2D materials.

**TABLE 2:** The advantages and disadvantages of different 2D materials

Materials	Advantages	Disadvantages	Ref.
Graphene	Excellent electrical conductivity and mechanical properties, a large specific surface area	The fabrication of graphene materials is costly	[29, 83]
MoS_2_	Excellent carrier mobility and thermal stability	Poor film uniformity	[44, 84]
AlOOH	High specific surface areas with abundant adsorption sites, numerous charged channel	Low electrical conductivity	[45, 85]
Layered double hydroxide	Inherently possess charged surfaces, high surface area, highly tunable interior architecture, nanochannels between layers	Low electrical conductivity	[40, 86]

Third, the self‐power devices based on 2D materials can only meet a little society's need for low‐energy‐consumption devices, such as transistors and sensors. In the future, the source of water in electricity generation should be more diverse. In daily life, in addition to the previously common atmospheric moisture, there are still many forms with the evaporation or flow of water waiting to be explored, such as the flow of dew, tides, moisture produced by running cars. What's more, the constant exploration of hydrovoltaic electricity generation will develop many neoteric applications for self‐powered devices in hot issues, such as artificial intelligence and medical technology. Self‐charging system may be another issue that need special attention. The energy generated by the self‐power device can be stored in capacitor which can provide a higher and more stable power. Future efforts should be focused on development of stable self‐charging systems. The self‐charging systems should possess some progressive features which meet the main need of society, such as long cycle, environment protection, and fast charging to make it better adapt to the development of society.

The recent progress has demonstrated that 2D materials for hydrovoltaic electricity generation applications will be a promising research direction in the future. However, as an emerging research field, there are still many opportunities and challenges that need to be faced. It is believed that with the continuous explorations by theorists and experimenters, the hydrovoltaic electricity generation based on 2D materials will be widely applied in the social life.

## CONFLICT OF INTEREST

The authors declare no conflict of interest.
